# The Impact of Malaria Parasites on Dendritic Cell–T Cell Interaction

**DOI:** 10.3389/fimmu.2020.01597

**Published:** 2020-07-24

**Authors:** Rowland S. Osii, Thomas D. Otto, Paul Garside, Francis M. Ndungu, James M. Brewer

**Affiliations:** ^1^Institute of Infection, Immunity & Inflammation, University of Glasgow, Glasgow, United Kingdom; ^2^KEMRI-CGMRC/Wellcome Trust Research Programme, Kilifi, Kenya; ^3^Centre for Tropical Medicine and Global Health, Nuffield Department of Medicine, University of Oxford, Oxford, United Kingdom

**Keywords:** dendritic cells, T cells, malaria, DC-T cell interaction, T helper cells, Tfh

## Abstract

Malaria is caused by apicomplexan parasites of the genus *Plasmodium*. While infection continues to pose a risk for the majority of the global population, the burden of disease mainly resides in Sub-Saharan Africa. Although immunity develops against disease, this requires years of persistent exposure and is not associated with protection against infection. Repeat infections occur due to the parasite's ability to disrupt or evade the host immune responses. However, despite many years of study, the mechanisms of this disruption remain unclear. Previous studies have demonstrated a parasite-induced failure in dendritic cell (DCs) function affecting the generation of helper T cell responses. These T cells fail to help B cell responses, reducing the production of antibodies that are necessary to control malaria infection. This review focuses on our current understanding of the effect of *Plasmodium* parasite on DC function, DC-T cell interaction, and T cell activation. A better understanding of how parasites disrupt DC-T cell interactions will lead to new targets and approaches to reinstate adaptive immune responses and enhance parasite immunity.

## Introduction

Malaria is caused by the *Plasmodium* parasite, which affects majority of the world's population. Annually, the disease causes ~228 million cases, resulting in 405,000 deaths. Africa accounts for about 93% of the reported cases and 94% of reported mortality cases occurring in children under the age of 5 (1). Residents in malaria endemic areas are susceptible to repeat malaria infection, with each infection resulting in modification of the hosts immune system. As well as affecting the host response to further infection (2), endemic malaria is also associated with weakened immunity to bystander infections and vaccines (3). Malaria infection has been shown to alter the phenotype and function of dendritic cells (4, 5) B cells (6, 7) and T cells (7–10) causing a disruption in the host immune response.

## *Plasmodium* Life Cycle

**Figure 1:**
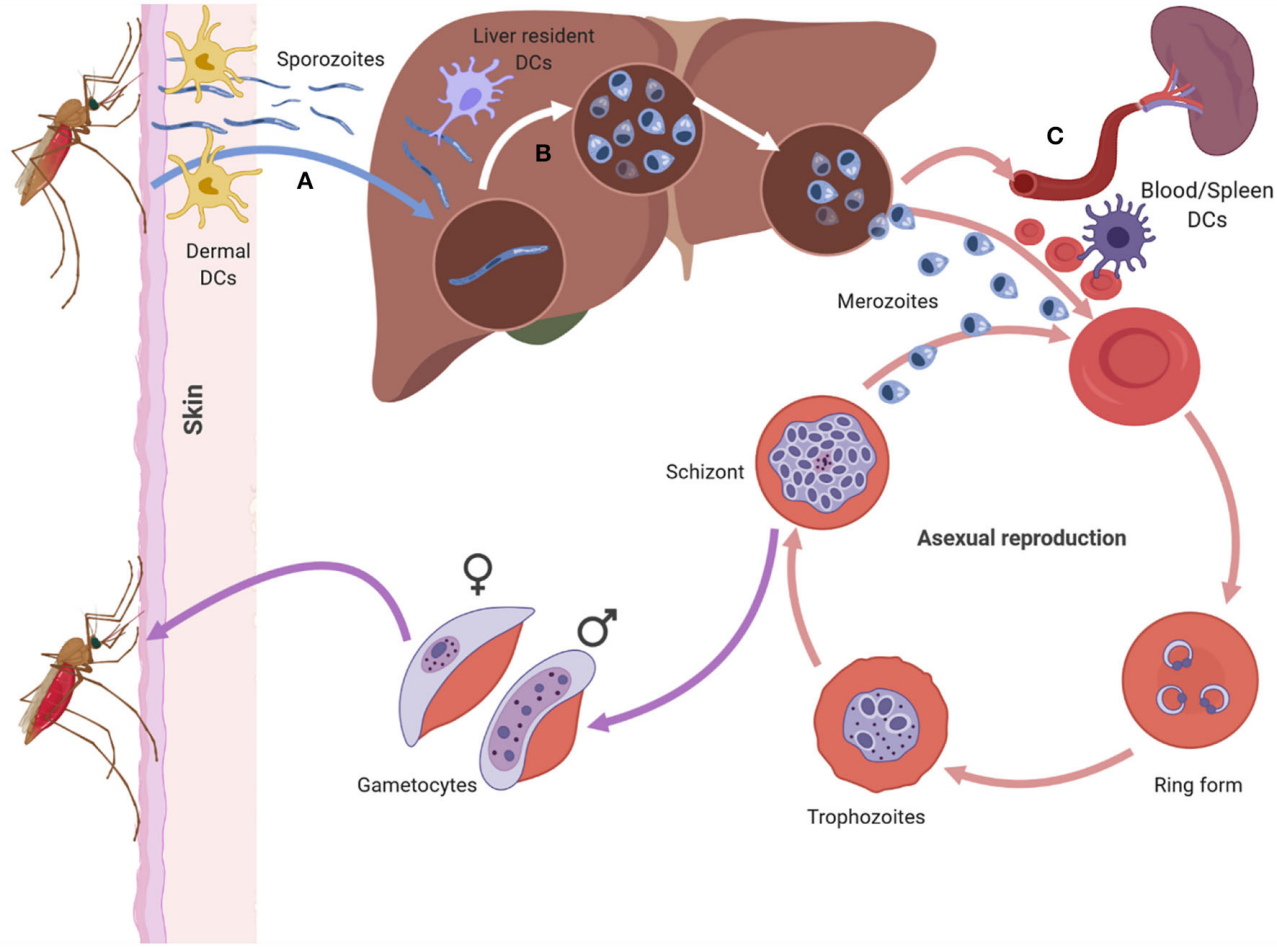
The asexual life cycle of Plasmodium parasite begins when an infected mosquito injects highly motile sporozoites into the skin of the host. The sporozorites enters the bloodstream and migrates to the liver, where it traverses multiple hepatocytes before infecting one. Inside the hepatocyte the sporozoite undergoes pre-erythrocytic schizogony forming merozoites that accumulate and bud off the hepatocyte in structures called merosomes. Merosomes enter the bloodstream and release merozoites which invade RBC, initiating the erythrocytic stage of asexual development. At this stage the parasite develops inside the RBC in distinct forms namely the ring, trophozoite, and schizont form. The schizont, lyses releasing merozoites into the blood stream which reinvade RBCs starting a fresh round of asexual development. After rounds of erythrocytic schizogony some of the asexual parasites develop into gametocytes and are taken up by a mosquito during a blood meal. Dendritic cells can interact with sporozoites in the dermis **(A)**, the liver **(B)** and the blood and spleen **(C)**. The DCs at each site encounter the parasite in its different forms (Figure was created using BioRender).

*Plasmodium* has a complex life cycle that occurs in two hosts; the female *Anopheles* mosquito (sexual reproductive stage) and a vertebrate host (asexual development stage). The latter begins when an infectious female *Anopheles* mosquito probes the dermis of a mammalian host as it takes a blood meal, releasing a highly motile form of the parasite, sporozoites, from its saliva (Figure 1A) (11, 12). Not all sporozoites manage to reach the blood vessel and those that remain in the dermis are either destroyed or drained into the lymphatics where the host's immune system eliminates them (13, 14). Those that manage to enter the bloodstream circulate and enter the liver through a process known as traversal, to gain access to a suitable hepatocyte (15, 16). Once inside a suitable hepatocyte, the sporozoite forms a parasitophorous vacuole (PV) and undergoes pre-erythrocytic schizogony, forming merozoites that accumulate within the parasitophorous vacuole and bud off the hepatocyte in structures called merosomes, clearing the liver of parasites (Figure 1B). The merosomes enter the bloodstream, releasing the encapsulated merozoites to infect red blood cells (RBCs) (17–19).

In the blood, the free merozoites attach to, and subsequently invade the RBC, initiating the erythrocytic stage of the parasite life cycle. Once inside the RBC, the merozoite matures in three morphologically distinct stages, namely the ring, trophozoite, and schizont stages. During the maturation stages the RBC undergoes a number of structural and functional changes that alter the architecture of the RBC membrane (Figure 1C) (20). Key amongst the structural changes is the expression of *Plasmodium falciparum* erythrocyte membrane protein 1 (PfEMP1), a vital parasite protein that is central to *P. falciparum* pathogenesis (21–23). PfEMP1 is expressed on the surface of parasite infected RBCs (iRBC) and enables iRBCs to sequester and cytoadhere to vascular endothelium, preventing their destruction in the spleen. Apart from the structural changes that occur to the RBC, the parasite also undergoes nuclear division producing merozoites that fill the PV (the schizont stage). The merozoites egress from the iRBC and invade other RBCs initiating another cycle for parasite replication.

After rounds of schizogony, some *P. falciparum* trophozoites commit to sexual development and form gametocytes. The gametocytes undergo five stages of maturation while being sequestered in the bone marrow. Only stage five gametocytes re-enter circulation and are taken up by a mosquito during a blood meal (24).

Interaction between DCs and *Plasmodium* parasite occurs at various points during the life cycle of the parasite in a human host (Figure 1). The parasite encounters DCs in the skin (Figure 1A) (13, 25), the liver (Figure 1B) (26, 27), and the blood and spleen (Figure 1C) (4). Tissue resident DCs in each of the sites can phagocytose parasite components and initiate specific immune responses to the parasite.

## Dendritic Cells

DCs are mononuclear phagocytic cells that are found in the blood, lymphoid organs and all tissues. They are the most effective professional antigen presenting cells in the body due to their ability to capture, process and present antigen on either major histocompatibility complex (MHC) class I or MCH class II molecules and activate naive CD8 or CD4 T cells (28, 29). DCs are central in initiating and regulating adaptive immune responses and act as a bridge between the innate and adaptive arms of the immune system. DCs differentiate from hematopoietic stem cells (HSC) (30) in the bone marrow to immature DCs, which circulate in blood and home to various peripheral tissues. Immature DCs recognize a range of danger signals such as pathogen-associated molecular patterns (PAMPS) which are found on pathogens and damage associated molecular patterns (DAMPS) which are released by injured host cells (31), through a number of pathogen recognition receptors (PRRs) (32, 33). Ligation of PRRs initiates DC phagocytosis, resulting in ingestion of the invading pathogen and initiation of DC maturation and migration into the lymph node where they present antigens to naive T cells (34). The maturation process results in increased expression of MHC surface molecule coupled with pathogen antigens and costimulatory molecules (CD80, CD86, and CD40), which are key in proliferation and differentiation of naive T cells into effector cells (35). DCs also secrete cytokines and chemokine that attract other immune cells to sights of infection/injury and influence the outcome of T and B cells responses (36).

DCs are lineage negative cells [that is they are defined by the exclusion of T cells (CD3), B Cells (CD19, CD20) natural killer cells (CD56), monocytes (CD14, CD16) and progenitor cells (CD34)] and express MHC class II (HLA-DR) and are broadly classified into either plasmacytoid DCs (pDCs) or conventional DCs (cDCs). In humans, pDCs are characterized by expression of CD123, CD303 (BDCA-2) and CD304 (37) and are known to produce large amounts of type I interferon in response to viruses (38). This is enabled by the high expression levels of toll-like receptor 7 (TLR7) and TLR9, which recognize nucleic acids from viruses, bacteria, and dead cells (39, 40). cDCs specialize in priming and presenting antigen to T cells. They can be further classified into cDC1 and cDC2. cDC1 express BDCA-3/CD141, CLEC9A, and XCR1 and have enhanced ability to cross present antigen (41) to CD8 T cells. cDC2 express BDCA-1/CD1c and have a wide variety of pattern recognition receptors (PRR's) and a good capacity to stimulate naive CD4 T cells but they have a poor ability to cross-present antigens to CD8 T cells compared with cDC1 (37, 40).

DCs are central in any immune response as they sense pathogens and initiate immune responses and are present at various sites during the life cycle of the *Plasmodium* parasite. As discussed later, the parasite's numerous immune evasion mechanisms interfere with DC function, thus altering downstream immune effector functions and the course of the disease.

## T Cells

T cells develop in the thymus from the common lymphoid progenitors which originate from bone marrow derived hematopoietic stem cells (42). After development and maturation, naiveT cells exit the thymus and enter circulation expressing either CD4 or CD8 and an antigen-recognizing T cell receptor (TCR) on their surface. The naive T cells home to secondary lymphoid organs (SLO) where they await a signal from DCs to become activated.

## CD8 T cells

Naive CD8 T Cells are activated by recognition of foreign or neoantigens presented by MHC class I molecules on DCs in the secondary lymphoid organs. Additional co-stimulatory signals and cytokines from DCs and/or CD4 T cells help in differentiation and clonal expansion of the T cells (43–46). The activated effector CD8 T cells migrate from the secondary lymphoid organs into circulation and identify their target cells which express cognate antigens on the cell surface bound to MHC class I. MHC class I is expressed on all nucleated cells except red blood cells. The target cells are killed by effector CD8 T cells through cell contact dependent cytolysis by releasing granzyme B and perforin (47–49). Perforin creates pores on the plasma membrane of the target cell; the pores allow granzyme B to enter the target cell and initiate apoptosis resulting in killing of infected cells. After clearing the invading pathogen, antigen specific effector CD8 T cells die off and a small number differentiate into memory CD8 T cells (45, 50).

Antigen specific CD8 T cells have been observed in the peripheral blood of residents from a malaria endemic area (51) and after vaccination of malaria naive individuals with irradiated sporozoites (52). In experimental mouse models of malaria, CD8 T cells specific for sporozoites antigens, liver stage antigens, and blood stage antigens were observed when mice were challenged with radiation attenuated sporozoites (53). It is believed that the priming of CD8 T cells against the pre-erythrocytic stages of *Plasmodium* occurs in the skin draining lymph nodes when sporozoites are injected into the skin by an infected mosquito (14, 54). These CD8 may offer protection against subsequent *Plasmodium* infections as incubation time in the liver offers a short window of opportunity for the CD8 T to mount an effective response.

## CD4 T cells

CD4 T cells, on the other hand, recognize antigens presented by MHC class II molecules, which are present on antigen presenting cells such as B cells, macrophages, and dendritic cells. CD4 T cells generally provide help to B cells in the germinal center enabling class switching and production of high-affinity antibodies (55). They also aid in CD8 T cell activation by licensing DCs (56–58) or directly signaling CD8 T cells via CD40 (59). They also secrete cytokines such interferon gamma (IFNγ), C-X-C motif ligand 9 (CXCL9), CXCL10 (60) interleukin-2 (IL-2) (61–63), and IL-21 (64) that are key in shaping immune responses. The diverse range of CD4 T cell functions are handled by distinct subsets of cells. The cytokine milieu in the microenvironment during CD4 T cell activation dictates the specific cytokine signaling networks and transcription factor activated for the differentiation of naive CD4 T cells into T cell subsets. The cytokines involved in CD4 T cell differentiation are produced by DCs and other innate immune cells, driving the cells to differentiate into either T-helper 1 (Th1), T-helper 2 (Th2), T-helper 17 (Th17), follicular helper T cell (Tfh), induced T-regulatory (iTreg), or the regulatory type 1 cells (Tr1).

Tfh cells have been a recent focus of interest in malaria immunology. Tfh cells express C-X-C motif receptor 5 (CXCR5) on their surface and are vital in the development of humoral immunity (55). Differentiation of CD4 T cells to Tfh is a multistep step process that first begins with DC interacting with a naive CD4 T cells in the T cells zone (**Figure 3**). This interaction results in the formation of pre-Tfh cells expressing CXCR5 that migrate to the T-B cell border of the SLO (65). At the T-B cell border and interfollicular zone, pre-Tfh interact with antigen specific B cells to initiate the B cell dependent phase of Tfh differentiation, which is characterized by upregulation of transcription factor B cell lymphoma 6 (Bcl-6) (66) and commits the Tfh lineage. After events at the T-B cell border, the Tfh migrates into the follicle and interacts with B cells forming germinal centers, where B cells undergo affinity maturation and heavy chain class switching, resulting in the production of high-affinity antibodies with enhanced effector functions (67). Tfh differentiation involves a number of cytokines such as IL-6, IL-21 (68), IL-12 (69), IL-27 (70), and TGF-β (71). These cytokines initiate signal transducer and activator of transcription 1 (STAT1), STAT3 (72) and STAT4 (73). The STATs upregulate the transcription factor B cell lymphoma 6 (Bcl-6), the master transcription factor in Tfh differentiation. Apart from cytokines, other signals required during differentiation of Tfh cells include the inducible costimulator (ICOS)- inducible costimulatory ligand (ICOSL) signaling (74, 75) and CD40-CD40L signaling.

**Figure 2:**
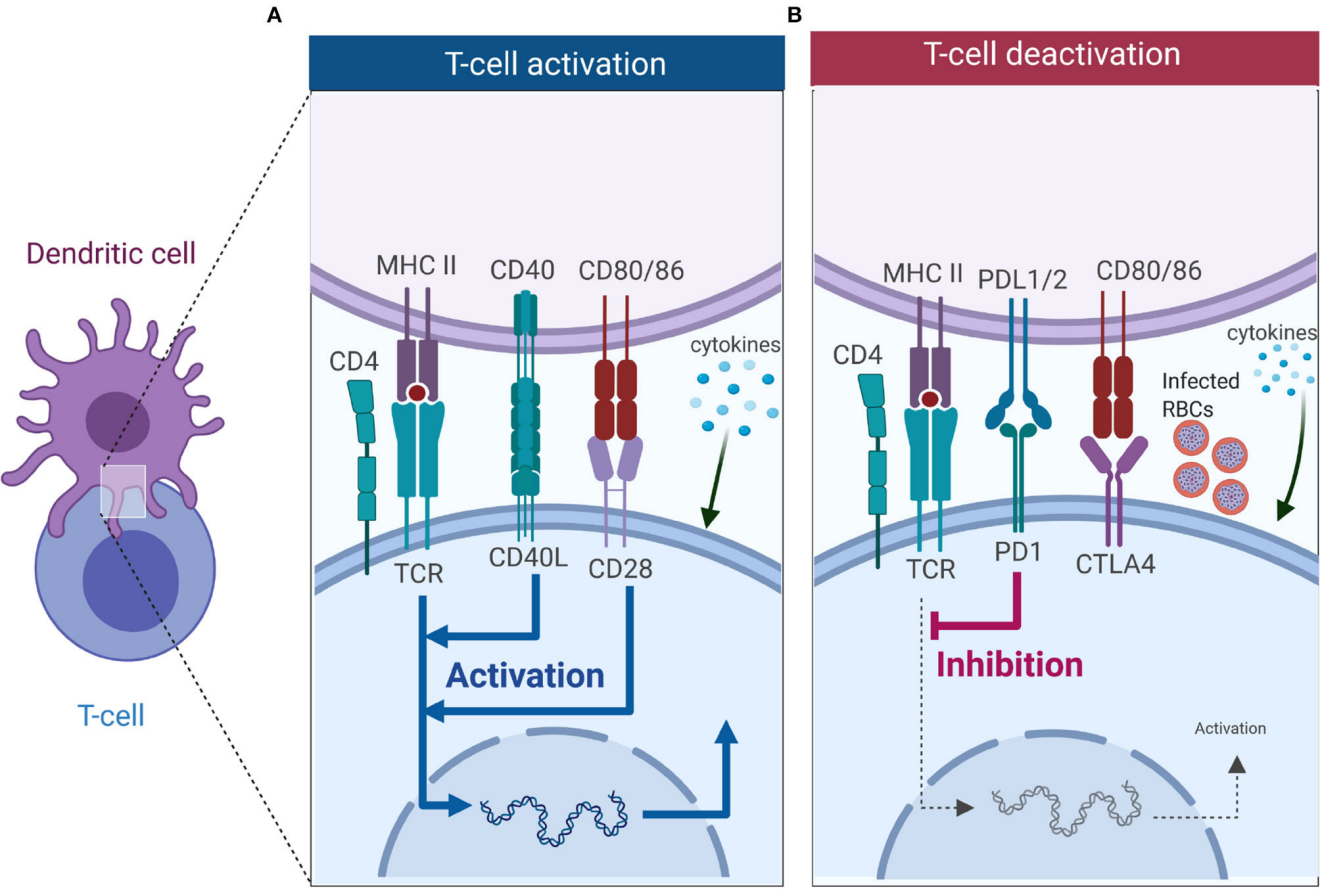
T cell activation or deactivation requires three signals from DCs. **(A)** T cell activation requires signal 1 in the form of TCR interacting with antigen-MHC complex which is key in innating downward signaling through ITAMs. Interaction of co-stimulatory molecules (interaction of CD40-CD40L, and CD80/86-CD28) form part of signal 2 as they work in tandem with TCR-antigen-MHC complex to enhance TCR signaling and initiate T cell proliferation. Signal 3 comes from DCs in the form of cytokines, and in CD4 T cells it is key in dictating which subset it will differentiate into. **(B)** Co-inhibitory molecules also form part of the second signal but unlike co-stimulatory molecules they inhibit TCR signaling, thus dampening immune responses. Interaction of PD-1 with PD-L1 inhibits T cell activation, while CTLA4 competes for binding with CD28 to CD80/86 and successful binding of CTLA4 to CD80/86 nullifies CD28-CD80/86 activation signal. Malaria has been shown to induce expression of PD-1, LAG3 and CTLA-4 on CD4 T cells, and this inhibits the activation signal from DCs (Figure was created using BioRender).

CD4 Tfh cells are essential for promoting antibody response that aid in resolving malaria infection (76, 77). In malaria infected humans and mice, Tfh cells adopt a Th1 like phenotype that expresses Tbet+ PD-1+, CXCR5+, CXCR3+, and secretes IFNγ (77, 78). This Tfh phenotype does not provide adequate help to B cells resulting in suboptimal antibody responses. Dysfunctional DCs that are induced by malaria may play a role in initiating this Th1-like phenotype that skews humoral response (Figures 2D,E).

## The impact of *Plasmodium* on DC-T cell Interactions

Activation of T cells requires interaction with DCs, which provide three key signals (Figure 2). Signal 1 occurs when T cells recognize cognate peptide antigen presented on either MHC I or MHC II on the surface of DCs via their T cell receptor (TCR). MHC-TCR interactions trigger activation of the T cells and initiates downwards signaling through immunoreceptor tyrosine-based activation motifs (ITAMs) (79). Besides TCR-antigen-MHC complex, a second signal, the costimulatory signal, is required to initiate and sustain T cell activation and proliferation. Co-inhibitory molecules (immune checkpoints) also form part of the second signal, but they downregulate immune responses (Figure 2B) (80, 81). Key costimulatory molecules involved in T cell activation include CD28 (binds to CD80/86 on DCs), ICOS (binds to ICOSL on DC), OX40 (binds to OX40L on DCs), and CD40L (binds to CD40 on DCs), are key in T cell activation, differentiation and survival (Figure 2A). These costimulatory signals work in synergy with the TCR-antigen-MHC complex to enhance the activation of T cells. Co-inhibitory molecules such as cytotoxic T-lymphocyte-associated protein 4 [(CTLA-4), competes for binding to CD80/86 with CD28 on DC], and programmed cell death-1 [(PD-1), binds to PD-1L] work to suppress the activation signal from TCR-antigen-MHC complex (Figure 2B). Once the T cell has received TCR-antigen-MHC complex signaling together with adequate co-stimulation, it receives a third signal in the form of cytokines that are secreted by DCs. As mentioned above, cytokines are important in deciding the fate of CD4 T cell differentiation toward a particular subset. Subsets of CD4 T cells include Th1 type (CD4 T cells exposed to the cytokine IL-12), Th2 (IL-4), Th-17 (IL-6, IL-23), Tfh (IL6, IL21), and iTreg (TGF-β) (Figure 2).

**Figure 3:**
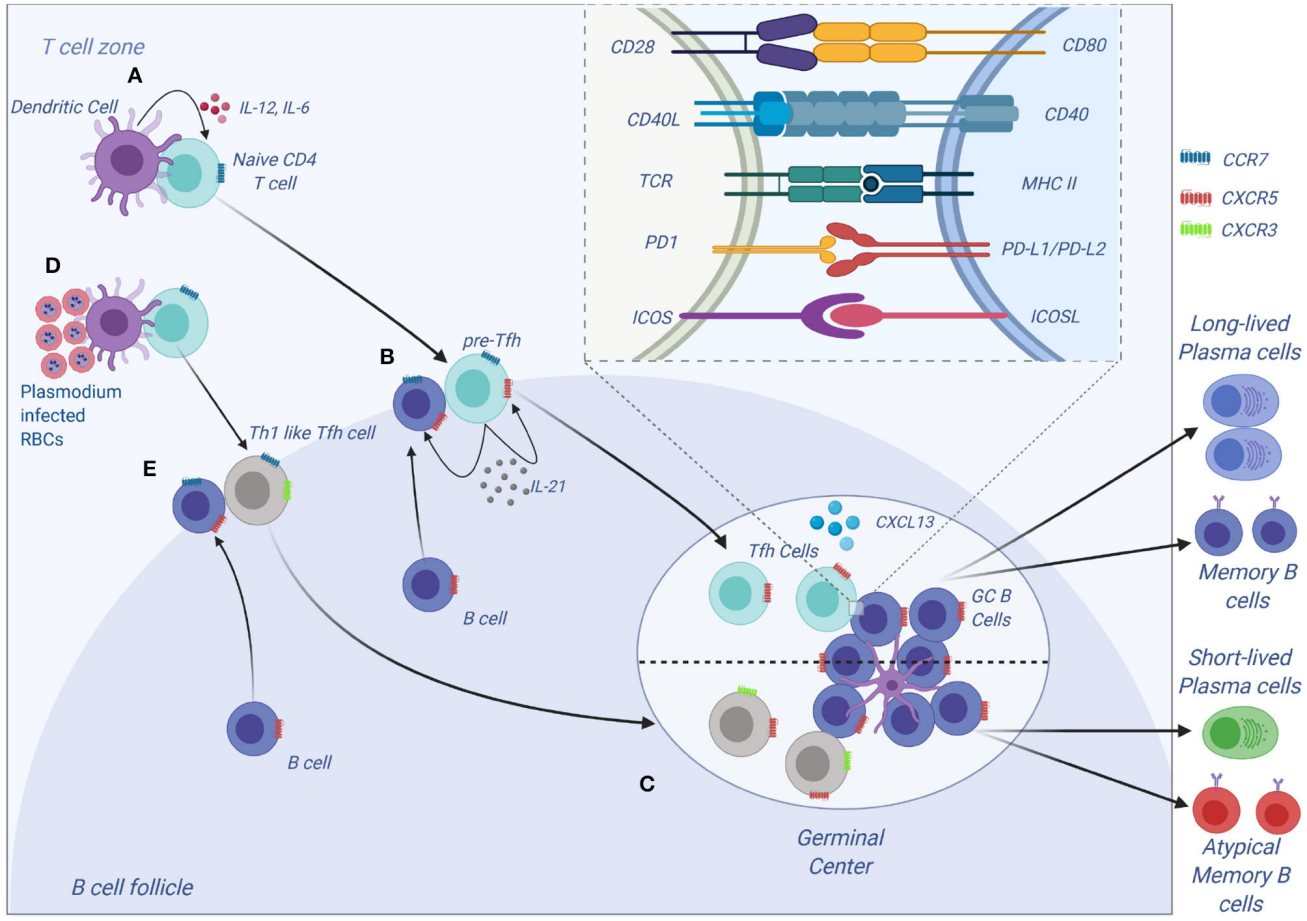
Tfh development and function. **(A)** Tfh differentiation begins when an activated DC primes a naïve CD4 T cells. Interaction between ICOS on CD4 and ICOS-L on DCs results in upregulation of Bcl-6 and CXCR5 on CD4 T cells. IL-12 and IL-6, help maintain expression of Bcl-6, and IL-12 induces upregulation of IL-21 which is essential for survival of the pre-Tfh cell. Initial CD4 T cell activation also results in upregulation of PD-1. The Bcl-6+CXCR5+ PD-1+ pre–Tfh cell then migrates to the T cell–B cell border. At the same time, antigen-activated B cells upregulate CCR7 and migrates from the B cell follicle to the T cell–B cell border. **(B)** At the T cell–B cell border, the pre-Tfh cell engages with the B cell. Interaction between antigen-MHC on B cells with TCR on pre-Tfh, and ICOSL (B cell) with ICOS (pre-Tfh cell), fully commits the cell to Tfh lineage. This results in upregulation and maintenance of Bcl-6, SAP, and CXCR5, while downregulating CCR7 on T cells. **(C)** The Tfh and B cells move deeper into the B cell follicle forming GCs. Tfh cells in the GCs promotes B-cell maturation, class switching and affinity maturation via the cytokines, IL-21 and IL-4, and the molecules, CD40L and PD-1. Both Tfh and GC B-cell are necessary for generation of B-cell memory and long-lived plasma cells. **(D,E)** *Plasmodium*-induces polarization of T follicular helper (Tfh) cells to Th1 like phenotytpe that expresses Tbet PD-1, CXCR5, CXCR3 and contributes to the inefficient acquisition of humoral immunity to malaria. Malaria infection in mice and humans induces secretion of Th1-polarizing cytokine that drive the activation of Th1-like Tfh cells that exhibit impaired B cell helper function, thus contributing to germinal center dysfunction and suboptimal antibody responses (Figure was created using BioRender).

Tfh differentiation is a multistep process that requires signal 1 in the form of antigen presented on MHC II by DCs (Figure 3). This interaction occurs at the T cell zone and involves the costimulatory molecules CD80, CD86, and inducible costimulatory ligand (ICOSL) on DC that interact with CD28 and ICOS to generate signal 2 in T cells. The CD28-CD80/86 interaction results in the upregulation of ICOS on T cells that interacts with ICOSL on DCs. The cytokine (signal 3) produced by DCs that helps in the initial process of Tfh differentiation is IL-12 (82). A combination of CD28-mediated signaling on T cells and IL-12 is adequate to upregulate the expression of Bcl-6, IL-12 also induces IL-21 production in T cells, which acts in an autocrine manner to ensure growth and survival of pre-Tfh. Bcl6 expression upregulates CXCR5 expression allowing the pre-Tfh cells to migrate to the T cell-B cell zone (83). At this zone, the Tfh cell interacts with B via ICOS-ICOSL committing the cell to the Tfh lineage and further upregulating CXCR5 and SAP (67). The CXCR5 and SAP expressing Tfh cells then move into the B cell follicle and form stable, long-lasting interactions with B cells forming germinal center where Tfh cells aid in class switching and generation of long-lived plasma cells that secrete high-affinity antibodies. Germinal center Tfh cells are also involved in the formation of long-lived plasma cells and memory B cells (84, 85).

## *P. falciparum* Immune Evasion and Suppression of Immunity

*P. falciparum* is equipped with multiple mechanisms which it uses to evade the host's immune system. These mechanisms include antigenic variation of surface antigens (VSA) expressed on iRBCs such as PfEMP1 which is encoded by the *var* genes (21), sub-telomeric variable open reading frame (STEVOR) encoded by the *stevor* genes (86, 87) and repetitive interspersed repeats (RIFIN) encoded by the *rif* genes (88, 89). Antigenic variation of VSAs normally occurs when the parasite is under intense immune pressure from the host in order to avoid recognition by various immune cells (90, 91). The expression of different VSAs on iRBCs allows the parasite to establish new infections (92). VSAs are key in sequestration and cytoadherence of maturing parasites (trophozoite and schizonts) and rosetting (93, 94). Merozoite surface protein (MSP) polymorphism (95–97) and complement evasion by surface proteins PfMSP3.1 (98), Pf92 (99), and PfGAP50 (100) expressed on merozoites and gametes are other mechanisms used by the parasite to escape elimination by the immune system.

Apart from immune evasion, ongoing *Plasmodium* infections have been shown to reduce immunogenicity of vaccines in children. Antibody responses to *Salmonella typhi* and tetanus vaccines were greatly reduced in malaria infected children compared to healthy control and children with other acute illnesses (3). Adults with previous exposure to *P. falciparum*, showed no response to malaria antigen, regardless of disease severity, and reduced response to non-specific antigens (2). Infection of influenza-immune mice with *P. chabaudi* resulted in a decrease in influenza specific antibodies and plasma cells resulting in a loss of protective immunity against influenza (101), which recovered several weeks after parasite clearance. This indicates that malaria infections somehow suppress immune function by interfering with the development of adaptive immunity. Ongoing malaria infection reduces immunogenicity to heterologous vaccines and malaria derived antigens. The exact mechanism used to induce this suppression is yet to be uncovered.

The suppression of immune function seen in malaria infection could be attributed to DC/iRBC interaction which alter the maturation state and function of DC in both humans (4, 102, 103) and mice (8, 104). DCs exposed to iRBC *in vitro* and *in vivo* have reduced expression of MHC on the surface and are unable to form stable interactions with CD4 helper T cells (104). The DCs also downregulate key costimulatory molecules, such as CD86, CD80, CD40, and secrete IL-10 (105), providing a suppressive environment for CD4 T cell development. This hampers their ability to activate naive CD4 T cells and a failure to generate Tfh cells that are critical in the formation of germinal center and generation of protective antibodies against malaria infection (77, 104). In contrast, other *in vitro* studies have shown that DCs exposed to iRBC successfully activate T cells, but induce their polarization toward a Th1 phenotype that inhibits commitment to Tfh cell linage, thus affecting humoral responses (103, 106).

## What Happens to DCs During a *Plasmodium* infection?

During the *Plasmodium* parasite life cycle, different forms of the parasite interact with resident DCs in various organs as it establishes infection. Sporozoites from infectious mosquitoes that are injected into the dermis interact with resident DCs in the skin (107). The sporozoites reach the liver interact with Kupffer cells, hepatocyte, and liver sinusoidal endothelial cells and resident DCs in the liver (108). The blood stage of the parasite interacts with DCs in the blood (4) and spleen (109–111).

## DC Interaction with *Plasmodium* Sporozoites in the Skin and Liver

The skin is the first point sporozoites encounter DC as they are inoculated by infected mosquitoes (Figure 1A). Studies conducted in mice have shown that only a small percentage of inoculated sporozoites leave the site of injection as most end up trapped in the dermis or enter the lymphatic system rather than the blood vessel (13). Other sporozoites infect keratinocytes, hair follicles, and develop into exoerythrocytic forms of the parasite (112). Sporozoites that are trapped in the dermis are phagocytosed by resident DCs which migrate to the skin-draining lymph node and can prime CD4 (113, 114) and CD8 (14) T cell responses. The immune response toward the sporozoite stage of the parasite may protect against subsequent challenges from infected mosquitoes (113).

The sporozoites that mange to enter blood circulation move to the liver and must traverse the sinusoidal barrier to access hepatocytes (Figure 1B) (115). The liver environment is tolerogenic due to the presence of IL-10 and TGF-β which are secreted by Kupffer cells (KC) and liver sinusoidal endothelial cells (LSEC) (116). These cytokines reduce expression levels of MHC class II and costimulatory molecules on the surface of liver resident DCs compared to resident DCs in lymphoid organs and those circulating in the blood (117) thus reducing their capability to activate T cells (118, 119). The tolerogenic environment of the liver could play a role in sporozoite immune evasion as DCs and other immune cells in the liver act to suppress adaptive immune responses which would lead to the elimination of sporozoites (116, 120).

Apart from DCs, the liver has other potential APCs that can present antigens to the adaptive immune system; this includes Kupffer cells (KC), liver sinusoidal endothelial cells (LSEC), and hepatocytes. LSECs are scavenger cells that express MHC class I and II molecules, low levels of CD86, and the adhesion molecules ICAM-1, VCAM-1, and dendritic cell specific intercellular adhesion molecule3-grabbing non-integrin (DC-SIGN). In mice, these cells have the ability to cross present antigens in the liver and activate CD8 T cells, but the T cells are generally tolerized due to the secretion of IL10 and PGE2 by LSECs (121, 122). KCs are resident tissue macrophages found in the liver that express MHC class I and class II molecules, ICAM-1, CD86, CD80, and can activate naive CD4 and CD8 T cells *in vitro* (123, 124). The role of KC as an APC is controversial as *in vitro* experiments show that they inhibit T cell activation by secreting IL-10 (125), but activation of KCs via TLR3 increased the expression of MHC class II and their APC function (126). Kuniyasu et al. (127) showed that the liver had the ability to retain adoptively transferred T cells. The T cells proliferated and expanded in the liver, but the expansion was followed by apoptosis, which was initiated by KCs (127). It was later shown that KCs induce T cell apoptosis via the FAS-FAS-L signaling pathway (128).

Hepatocytes express MHC class I and ICAM-1 in their steady state and during inflammation they have been shown to express MHC class II CD40L, CD80 and CD86 and are capable of activating CD8 T cells (129). Their role in generation of malaria liver immunity has been controversial with different studies using mouse models drawing different conclusions of their role in the generation of pre-erythrocytic immunity. Intrasplenic injection of parasite infected hepatocytes in mice resulted in T cell mediated immunity against *P. yoelii* and *P. berghei* infections (26), thus showing that hepatocytes are capable of activating T cells. Another study demonstrated that parasite infected hepatocytes undergo apoptosis, thus providing liver DCs with a source of *Plasmodium* antigens for initiating the adaptive immune response (130). This idea has been challenged and it has been suggested that DCs could obtain *Plasmodium* antigens directly from viable infected hepatocytes. This is supported by the fact that DCs have the ability to acquire antigens from other live cells and cross present to CD8 T cells (131).

Chakravarty et al. (14) showed that cross presentation of *Plasmodium* antigens by DCs was key in CD8 T cells activation and this occurred in the skin draining lymph node, not in the liver, and the activated T cells recirculated to the liver (14). Indicating that DCs in the skin that encounter sporozoite play a crucial role in generating T cell mediated liver immunity. Recently Kurup et al. (27) showed that during a malaria infection, a subset of monocyte derived CD11c+ APC infiltrate the liver after hepatocyte infection by *Plasmodium* parasite and acquire *Plasmodium* antigens. The monocyte derived CD11c+ APC present the antigens to naive CD8 T cells in the liver draining lymph node, priming them and initiating T cell mediated immunity against *Plasmodium* infection (27).

While there are still some gaps into how the generation of liver immunity against *Plasmodium* infection is acquired, it is clear that APCs, especially DCs, play a central role. Hepatocytes may play a part in the generation of liver immunity by providing parasite antigens to DCs but the exact mechanism of this is yet to be uncovered. A better understanding of DC and hepatocyte involvement in the generation of liver immunity is required and also the roles played by KCs and LSECs. The use of humanized mice might provide an opportunity to further investigate skin and liver immunity against *P. falciparum* (132, 133).

## DC Interaction with *Plasmodium* during the Blood Stage of Malaria

The blood stage of the malaria parasite life cycle provides several opportunities for DC in the blood and spleen to interact with infected RBC (Figure 1C). This stage requires remodeling of the RBC to enable the parasite to survive (134) and results in the expression of parasite antigens on the RBC surface. These antigens, in particular PfEMP, play a key role in immune evasion and vascular sequestration/cytoadherence to avoid splenic clearance (21, 135). It has been suggested that PfEMP1 may be involved in modulation of DC function via interaction with CD36 (4, 136).

Maturation of iRBCs (schizont stage) results in lysis of the iRBCs, releasing merozoites into circulation and the contents of the PV such as the parasites digestive vacuole which contains hemozoin and waste products. The free merozoites have a short window to invade new RBCs (137) and those that fail to invade remain in circulation where they are phagocytosed by immune cells or cleared in the spleen. Parasite waste products and hemozoin do interact with DCs but their overall effect on DC function is contradictory. The effect of hemozoin on DCs has yielded varying results with some studies showing that hemozoin is capable of activating DCs (138) while others showed that DC maturation and function was inhibited by hemozoin (8, 139). The varying results could be due to the different methods that were used to generate hemozoin with contamination by parasite DNA being a potential confounding factor (140).

Overall, the blood stage has an abundance of parasite antigens that DCs can use to mount an immune response. However various immune evasion mechanisms, such as antigenic variation of VSAs (141, 142) and sequestration of mature schizont and trophozoites in blood capillaries (143) thus avoiding splenic clearance (144, 145) the slow acquisition of immunity. DCs at this stage are critical in maintaining an immunological balance between parasite burden and a sufficient immune response. Immune evasion by the parasite could cause an increase in parasite burden resulting in severe pathology, while an excessive and uncontrolled immune response may lead to the development of a severe life threating cerebral malaria (146–148).

Studies of blood stage infections with DC have largely employed DCs prepared from peripheral blood monocytes or isolated from peripheral blood of uninfected individuals (4, 102). Fewer studies have analyzed the phenotype and function of peripheral blood DCs from individuals who are currently undergoing a malaria episode (103, 149, 150). In this context, *in vivo* mouse models of malaria have been particularly helpful to understand the tissue responses of DC, for example splenic DC and allow temporal analysis of how *Plasmodium* infection changes DC phenotype (8, 104).

## *In vitro* DC interaction with Plasmodium

*In vitro* studies have been used to identify the mechanisms used by the parasite to modulate DC function. These studies have either used human monocyte derived dendritic cells (moDCs) or bona fide DCs to assess DC- *P. falciparum* interactions.

Urban et al. (4) showed that when moDCs were co-cultured with *P. falciparum* iRBC, at a ratio of 1:100, and later stimulated with lipopolysaccharide (LPS), exhibited a decreased expression of key maturation markers (CD40, CD80, CD86, and CD83) (4). Once moDCs were exposed to iRBC, they lacked the capacity to activate allogeneic T cells (4). This modulation of DC maturation may result from an interaction between CD36 on DCs with PfEMP-1 on iRBC (105). A subsequent study found that a ratio of 1 DC: 100 iRBC inhibited LPS induced moDCs activation, cytokine production, and allogeneic T cell activation regardless of CD36-binding with iRBC (102). The high ratio of DC to iRBC coincided with an increase in apoptotic and necrotic cells, which was observed in both PfEMP1-deficient iRBCs and PfEMP1 expressing iRBCs, this could account for the failure of DCs to respond (102). At low iRBC to moDC ratio (10:1), moDCs made a modest response to LPS induced maturation and retained their ability to secrete cytokines and activate T cells (102). Elliot et al. (102) were unable to point out the mechanism used by *P. falciparum* to modulate moDC function, although they found that hemozoin, from iRBC lysate, did not inhibit LPS maturation of moDCs (102). The studies show that a dose-dependant relationship exists between iRBC and moDCs inhibition and dose range experiments are an essential part of ensuring experimental reproducibility in the future.

Another study found that at a low ratio of 10 iRBC per moDCs did not trigger the upregulation of HLA-DR, CD83, or CCR7 on moDCS (151), contradicting the study by Elliot et al. (102). At a ratio of 100 iRBCS per moDCs, moDCs were able to secrete IL-1β, IL-6, IL-10, TNF-α, and upregulate the chemokine receptor CXCR4 (151). Exposure of moDCs to schizont lysate resulted in an increase in the expression levels of CD86 while CD80 and HLA-DR levels remained unaffected even at high concentration of schizont extract (106). Exposure to schizont lysate, followed by LPS stimulation, did not affect the maturation of moDCs. The schizont lysate exposed moDCs maintained their ability to differentiate allogeneic T cells into Th1 and regulatory T cells (Treg) that secrete large amounts of IFN-γ. Additionally, the generated Tregs also secreted IL-10 and TGF-β (106).

The different *in vitro* studies looking at the effect of *P. falciparum* on moDCs have yielded varying results. This could be attributed to the use of the *Plasmodium* parasite at different stages of development in the RBC. Another explanation could be that the studies used different experimental methods in the isolation of the *Plasmodium* infected red blood cells and in the generation of moDCs.

Few studies have examined the effect of *P. falciparum* on cDCs and pDCs due to their low numbers in peripheral blood. One study examined the effect of *P. falciparum* on cDC2 and pDCs (103). The co-culture of cDC2 with *P. falciparum* at a ratio of 1:3 resulted in the upregulation of maturation markers (CD80, CD86, CD40, and HLA-DR) and inflammatory chemokines CCL2, CXCL9 and CXCL10 but did not induce secretion of inflammatory cytokines. Exposure of cDC2 to iRBC did not inhibit cytokine secretion in response to LPS, which was contrary to what was observed with moDCS (103). The low ratio of iRBC to DC may account for this observation as the study did not use a higher ratio of iRBC to cDC2. The cDC2 exposed to iRBC maintained their ability to present antigens and activate naive T cells to polarize them toward a Tfh1 phenotype that secretes IFN-γ (103). The study also found that crosstalk between pDCs and cDC2 was important in shaping immune responses against malaria. The co-culture of pDCs and cDCs resulted in the upregulation of HLA-DR, CD86, and CD40 on pDCs and CD80 and CD86 on cDC2. There was also an increase in the secretion of interferon alpha (IFN-α) by pDCs and chemokines CXCL9 and CXCL10 by cDC2. This cross-talk between these two DCs was contact dependent, suggesting cell to cell interaction is necessary to initiate chemokine secretion (103). The study highlighted the importance of cell to cell interaction which is crucial in trying to understand immune responses in malaria.

In mouse studies using bone marrow derived dendritic cells (BMDCs), *P. chabaudi* schizonts were shown to be able to activate BMDCs to produce the pro-inflammatory cytokines IL-12 and TNF-α. The *P. chabaudi* exposed DCs did not inhibit LPS activation, contrary to what was observed with *P. falciparum* exposed human DCs (152).

## *Ex vivo* DC interaction with *Plasmodium*

A number of studies have compared peripheral blood DCs in varying malaria transmission settings and different at-risk groups. In Kenya, children hospitalized with either mild or severe malaria were found to have a lower number of DC expressing HLA-DR and a lower number of circulating DCs compared with healthy children (149). A follow up study revealed that the expression levels of HLA-DR was reduced on monocytes and cDC but not on pDC and that DC modulation continued during convalescence. An increase in the frequency of BDCA3+ cDC1 in the peripheral circulation was also observed during the course of the malaria infection (153).

A similar study was conducted in Mali looking at the function of DCs in children with severe malaria from the Dogon and Fulani community. The two communities reside in the same geographical region and are exposed to the same intensity of *P. falciparum* transmission yet the Fulani are less suspectable to *P. falciparum* infection (154). DCs from malaria infected children of the Dogon community expressed lower levels of HLA-DR and CD86 on their DCs, while the frequency of BDCA-2+ pDCs and BDCA-3+ cDC1 increased compared to uninfected counterparts. Infected children from the Fulani community exhibited higher levels of HLA-DR and CD86 on their DCs but had a lower number of circulating BDCA-2+ pDCs and BDCA-3+ cDC1 compared to their uninfected counterparts (150). The study also showed that infected children from the Fulani community retained their ability to produce IFN-γ after their PBMC were stimulated with specific TLR ligands at levels that were similar to those of uninfected children. The Dogon children, on the other hand, had low levels of cytokine produced due to TLR impairment which increased parasite burden and development of malaria symptoms (150). This showed that *P. falciparum* infection resulted in altered DC activation with reduced response to TLR agonists in Dogon children, while in the Fulani children, DC activation and TLR responses were unaffected.

The increase in the number of circulating BDCA-2+ pDCs and BDCA-3+ cDC1 during malaria infection has been attributed to an increase in the amounts of FMS-like tyrosine kinase 3 (Flt3) ligand (Flt3-L) (155). Flt3 is highly expressed on hematopoietic progenitor cells, but the expression is lost as cells commit to lymphoid and myeloid progenitor cells, which gives rise to the various cell lineages but its expression on DCs remains. Flt3 receptor tyrosine kinase and its ligand Flt3-L are known to be key in the development of dendritic cells and maintenance of their numbers (156, 157). Flt3-L production increases during a malaria episode as mast cells become activated and release membrane bound Flt3-L into circulation resulting in an increase in the number of pDCs and CD1c (155).

A few studies have looked at the function of DCs in adults during a malaria episode. A study in Thailand found that adults with both severe and mild malaria had a decreased number of TLR2 expressing cDCs circulating in the periphery and a lower surface expression of TLR9 on pDCs but an increase in the surface expression of TLR2 on cDCs compared with healthy controls (158). There was also a marked reduction in the number of circulating pDCs, this could be attributed to their migration to the secondary lymph nodes, and an increase in serum levels of IFN-α (159). A study conducted in Papua found that adults with acute *P. falciparum* malaria had a reduced number of circulating pDCs and cDCs, but higher numbers of immature DCs that were HLA-DR+CD11c–CD123– (5). Interestingly both pDCs and cDCs from infected participants were apoptotic as seen by Annexin-V binding. The DCs also expressed low levels of HLA-DR and costimulatory molecules and were unable to adequately capture antigen, resulting in reduced ability to prime naive CD4 T cells (5). These studies are therefore consistent with a role for malaria infection in reducing the number of circulating DCs and their function in antigen presentation and T cell activation.

Controlled human infection model (CHMI) have also been used to assess the function of BDCA-1+ cDC2 and pDCs at varying doses of *P. falciparum* (160). Healthy volunteers were enrolled into two cohorts; one cohort was inoculated with 150 iRBCs and the other 1,800 iRBCs, participants were treated once parasitaemia reached ≥ 1,000 parasites/ml (160, 161). The expression levels of HLA-DR on BDCA-1+ cDC2 and pDCs in both cohorts were significantly reduced at peak parasitaemia and this effect was still evident on BDCA-1+ cDC2 24 h after anti-malarial treatment. The cohort inoculated with a higher dose of iRBC had a reduced number of circulating BDCA-1+ cDC2 which was attributed to apoptosis of the DCs during the course of the infection, this was evident by the upregulation of caspase-3 (160). The BDCA-1+ cDC2 from this cohort had a defective phagocytic capacity and there was a positive association between HLA-DR expression and phagocytic capacity (160). pDCS on the other hand expressed low levels of CD123 at peak parasitaemia in both cohorts which persisted 24 h after anti-malarial treatment. The number of pDCs in circulation significantly reduced in the s iRBC cohort, this was due to apoptosis of pDCs during the course of infection (161). At peak parasitaemia DCs from the 1,800 iRBC cohort were restimulated *ex vivo* with TLR ligands and their response measures. On re-stimulation with TLR1/2, TLR4, and TLR7, BDCA-1+ cDC2 failed to upregulate HLA-DR and CD86 but increased TNF secretion (160). While re-stimulation of pDCS with TLR7 and TLR9 resulted in upregulation of HLA-DR, CD123, CD86 on their surface and an increased secretion of IFN-α (161). This shows that malaria infection in naive individuals results in impairment of cDC function but not pDCs function. Indicating that pDCs may play a role during malaria infection and further studies are needed to deduce its role. The altered BDCA-1+ cDC2 also contributed to hampering effector T cells functions, allowing an increase of parasite burden (160).

The various studies above have shown that DC phenotype is altered during a malaria episode resulting in impaired ability to upregulate HLA-DR and the costimulatory molecules CD86 (150, 153, 160). This altered DC phenotype has a reduced phagocytic capacity which impairs its ability to process antigens (160) and adequately stimulate allogeneic T cells (5, 153). The parasite also modulates TLR signaling thereby affecting cytokine secretion (150, 160) resulting in severe pathology. In children, there seems to be a notable increase in the number of circulating BDCA-3+ cDC1s during a malaria episode (150, 153, 155), which was attributed to increases in serum levels of Flt3-L (155), but this effect was not observed in children from Papua (162). In both children and adults, there was a decrease in the number of circulating DCs which was attributed to increased DC apoptosis (5, 159, 161) but also increased DC migration to secondary lymphoid organs may also play a role in reduction on peripheral blood DC numbers. The decrease in peripheral numbers of DCs also corresponded with an increase in IL10 and TNF-α (5, 149, 153), which may play a role in DC loss of function and suppression of T cell function. In these studies, DC function was altered regardless of the severity of malaria infection. The DC phenotype seen in the acute infection in the CHMI study (160), was similar to those seen in naturally exposed individuals, and repeated infection, in naturally exposed individuals, could lead to sustained downregulation of DC function that may impact negatively on the immunity of an individual.

## *In vivo* Mouse Models of Malaria

Mouse models have been extensively used to study DC-*Plasmodium* interaction. *In vitro* interaction of *P. chabaudi* schizonts with mouse bone marrow derived DCs resulted in an increase in the secretion of tumor necrosis factor-α(TNF-α), IL-6, and IL-12p40 and IL-12p70 (152). In mice injected with *P. chabaudi*, DCs had fully functional cytokine production 6 days after challenge with *Plasmodium* parasite (163). Further studies demonstrated DCs were able to upregulate co-stimulatory molecules CD40, CD54, CD86 (164) during acute infection, and were able to migrate into T cell areas in the spleen (165). Other studies with *P. chabaudi* show that during initial stages of murine erythrocyte infection, CD8+ DCs are activated by infected erythrocytes as they expressed high levels of MHC II and costimulatory molecules and initiated a Th1 type of response. This response is short lived as the CD8+ DCs undergo apoptosis and are soon replaced by CD8- DCs with lower expression levels of costimulatory molecules and MHC II (166).

Consistent with the studies above, Millington et al. (8) showed that DCs isolated from the spleen of mice 4 days after *P. chabaudi* infection were moderately activated as they upregulated surface expression of CD40, CD80, and CD86. However, during convalescence (days 12 and 21 post-infection), DC did not upregulate costimulatory molecules and were refractory to stimulation with LPS or CD40L. When mice infected with *P. chabaudi* were immunized with ovalbumin (OVA) antigen and LPS, they produced significantly lower levels of OVA-specific IgG compared with uninfected immunized mice, however, this effect was only seen when immunized at days 12 and 21 post infection (not day 4). Thus, initial malaria infection in mice does seem to cause DC activation; however DCs enter a refractory state in following the initial peak of parasitaemia. Similar to convalescent DCs, *in vitro* bone marrow derived DCs pre-exposed to *P. chabaudi* were unable to increase expression levels of MHC II and co-stimulatory molecules CD40, CD80, and CD86, and LPS stimulation of these DCs was unable to increase their expression (8).

Further work suggested that hemozoin could also modulate DC function which resulted in impairment of T cell and B cell function. Hemozoin treated DCs retained their capacity to process antigen and present them on MHC class II to naive CD4 T cells. Thus providing the essential signal 1 (peptide-MHC complex) via the T cell receptor (TCR) but these DCs were unable to form stable long lasting clusters with naive T cells, resulting in the generation of dysfunctional T cells (8, 104). These dysfunctional T cells failed to proliferate and produce adequate amount of effector cytokines (IL-2, IL-5, IL-10, IFNγ) (8), and were unable to migrate to B cell areas in the lymph nodes to aid in B cell proliferation and antibody production (8, 104). The short interactions and lack of large clustering observed are known to interfere with the generation of Tfh cells as long sustained DC-T cells interaction is required for commitment of naive CD4 T cells to Tfh cells (167). It is possible the dysfunctional DCs can lead to the generation of exhausted T cells, as a result of the short time of antigen presentation to the T cells in the absence of adequate co-stimulation. The dysfunctional T cells could also lead to the generation of atypical memory B cells which are normally associated with malaria episodes.

Dendritic cells have been shown to play a vital role in the survival of mice during a lethal infection with *P. yoelii*. Wykes et al. (168) showed that DCs from mice infected with non-lethal *P. yoelii* infection were fully functional APC and maintained their ability to stimulate T cells, unlike DCs from lethal *P. yoelii* infection which were not functional. DCs from mice infected with the non-lethal parasite were adoptively transferred into naive mice, which were then infected with lethal infection *P. yoelii*. These DCs were able to control parasitaemia and aid in survival of the mice by secreting IL-12 (168). This could in part explain the difference in malaria outcomes observed in natural infections.

## Downstream Effect of DC Dysregulation

Collectively, the studies above support the hypothesis that during the blood stage of *Plasmodium* infection, DC function is dysregulated resulting in phenotypically altered DCs that are unable to appropriately activate naive CD4 cells. Furthermore, the failure by naive CD4 T cells to differentiate into CD4 follicular helper T cells results in a failure of B cell help and reduced humoral immunity. The phenotype of the resulting T cell population is unclear; however, *P. falciparum* infection has been associated with increased expression of the T cell inhibitory receptor programmed cell death-1 (PD-1) on CD4 T cells. This was observed in a cohort of children in Mali (169) and Kenya (7), during an ongoing *P. falciparum* infection. Apart from an increase in the expression of PD-1 on CD4 T cells, *P. falciparum* infection was seen to drive an increase in the frequency of atypical memory B cells which was as a result of the exhausted T cell phenotype (7).

Butler et al. (169) using non-lethal *P. yoelii* infections, also showed that prolonged infection resulted in dysfunctional parasite specific CD4 T cells that expressed exhaustion markers PD-1 and lymphocyte-activation gene-3 (LAG-3) (169). These inhibitory ligands worked in synergy to inhibit T cell function during the *Plasmodium* infection. The ability of CD4 T cells to produce cytokines deteriorated with prolonged infection, while dual blockade of PD-1 and LAG-3 with monoclonal antibodies restored the number of parasite specific CD4 T cells and their ability to secrete cytokines. It also resulted in an increase in the number of CD4 Tfh cells and plasmablasts, thus improving the anti-parasite humoral response in *P. yoelii* infected mice (169). These studies show that that malaria induces T cell exhaustion and that PD-1 plays a role in the pathogenesis of malaria.

Apart from the upregulation of the T cell inhibitory receptors programmed cell death-1 (PD-1) and Lymphocyte Activation Gene 3 (LAG3), malaria infection upregulates the production of IFN-γ and IL-10 on CD4 T cells (170, 171). This creates a suppressive environment that polarizes CD4 T cells toward IFN-γ producing Th1 like lineage, suppressing induction of Th2 and Tfh, which are vital in B cell response. This polarization occurs after a single malaria episode and may affect subsequent parasite exposures

T cell exhaustion can be due to persistent antigen exposure, resulting in sustained TCR stimulation by dysfunction DCs, leading to sustained upregulation of PD-1 (172, 173). The inhibitory PD1 signal on T cells works by inhibiting downward signals from TCR and costimulatory molecules and initiates transcription of inhibitory genes (Figure 2B) (174–176). Cytokine signaling also plays a role in T cell exhaustion. Malaria induces secretion of IL-10 from DCs, providing an immunosuppressive environment that skews the development of CD4 T cells and dual blocking IL-10 and PD-1 signaling in mice restores T cell function (177). Transcriptional profile of exhausted T cells greatly varies from effector and memory T cells, indicating that exhaustion is a unique state of T cell differentiation (178–180), that is regulated by the master transcription factor TOX (181). The signaling pathways that lead to the differentiation of exhausted T cells and expression of TOX are yet to be known.

There are still gaps in our current understanding of the intracellular mechanism of PD1 signaling and what its target genes are. The molecular events initiated by downstream IL-10 signaling that shape T cell exhaustion are yet to be known.

## Conclusion

The population in malaria endemic areas are known to have a reduced immune response against vaccines (2, 3), and ongoing malaria infections in these individuals reduce pre-existing adaptive immune responses (101). The evidence presented above strongly indicates this is due to dysfunctional DCs that fail to prime effective T cell responses, thus affecting immune responses. There is a potential gap is in our understanding of the effect of antimalarial drugs on the phenotype, function and numbers of DCs and T cells. Whether antimalarial treatment restores DC-T interaction is an area of research is yet to be explored.

This information could address a significant public health challenge in administering malaria vaccines and other vaccines in malaria endemic areas. Malaria infection also induces CD4 T cell exhaustion, through upregulation of negative regulatory molecules such as PD-1 and LAG3 (7, 169), which dampen immune responses. How T cell exhaustion is induced in malaria is still unknown as there is no evidence in literature explaining how and where these cells arise, and if dysfunctional DCs play a role in this, and how the cytokine environment during a malaria episode influence T cell exhaustion. There is a need to better understand the interaction between DC-T cell, the cellular and molecular signals that are involved in the formation of this immune synapse and how malaria affects this interaction. This will aid in developing novel methods that will target the affected molecular pathways and restore DC-T cell interaction and function.

## Author Contributions

RO wrote the first draft of the manuscript, which was reviewed and edited by TO, FN, PG, and JB. All authors contributed to the article and approved the submitted version.

## Conflict of Interest

The authors declare that the research was conducted in the absence of any commercial or financial relationships that could be construed as a potential conflict of interest.
